# Transcriptomic and Functional Screens Reveal MicroRNAs That Modulate Prostate Cancer Metastasis

**DOI:** 10.3389/fonc.2020.00292

**Published:** 2020-03-13

**Authors:** Srinivasa R. Rao, Alison Howarth, Patrick Kratschmer, Ann E. Snaith, Clarence Yapp, Daniel Ebner, Freddie C. Hamdy, Claire M. Edwards

**Affiliations:** ^1^Nuffield Department of Surgical Sciences, University of Oxford, Oxford, United Kingdom; ^2^Nuffield Department of Medicine, Target Discovery Institute, University of Oxford, Oxford, United Kingdom; ^3^Nuffield Department of Orthopaedics, Rheumatology and Musculoskeletal Sciences, Botnar Research Centre, University of Oxford, Oxford, United Kingdom

**Keywords:** microRNA, prostate cancer, screening, EMT - epithelial to mesenchymal transition, morphological analysis, migration screening

## Abstract

Identifying new mechanisms that underlie the complex process of metastasis is vital to combat this fatal step in prostate cancer (PCa) progression. Small non-coding RNAs are emerging as important regulators of tumor cell biology. Here we take an integrative approach to elucidate the contribution of microRNAs to metastatic progression, combining transcriptomic analysis with functional screens for migration and morphology. We developed high-content microscopy, high-throughput functional screens for migration and morphology in PCa cells using a microRNA library. RNA-Seq analysis of paired epithelial and mesenchymal PCa cells identified differential expression of 200 microRNAs. Data integration identified two microRNAs that inhibited migration, induced an epithelial-like morphology and were increased in epithelial PCa cells. An overrepresentation of the AAGUGC seed sequence was detected in all three datasets. Analysis of published datasets of patients with PCa identified microRNAs of clinical relevance. The integration of high-throughput functional and expression analyses identifies microRNAs with clinical significance that modulate metastatic behavior in PCa.

## Introduction

Metastasis is responsible for >90% of human cancer related-deaths, and a comprehensive understanding of the cellular and molecular control of metastatic spread is imperative in order to develop new approaches to combat this fatal stage (1). The metastatic cascade is a multistep process that begins with tumor cells at the primary site undergoing morphological changes, facilitating their migration to distant sites for subsequent colonization. In advanced prostate cancer, malignant epithelial cells escape the prostate capsule, and can seed distant tissues like lymph nodes, bone marrow and adrenal glands (2). They do this by migrating and invading through several barriers, including the basement membrane, connective tissue of the prostatic capsule and blood vessel walls. Whereas, localized prostate cancer has a good prognosis, once prostate cancer has metastasized to distant sites, the disease is ultimately fatal and treatment is largely palliative.

MicroRNAs are short non-coding RNAs that are 20–23 nucleotides in length which cause translational repression or mRNA degradation by binding to cognate regions in the 3′ untranslated regions (UTRs) of messenger RNA. A 6–8 nucleotide sequence at the 5' end of the microRNA, called the “seed” region, is crucial for the majority of miRNA:mRNA interactions. MicroRNAs have emerged as key regulatory molecules in multiple facets of tumor cell behavior, and migration is no exception. A number of studies have underlined the importance of microRNAs in prostate cancer pathogenesis, including the control of cellular morphology and migration in prostate cancer cells (3, 4). However, these previous studies utilize a candidate-based approach to investigate the role of microRNAs, which could potentially miss out on identifying crucial players. Hence, we aimed to employ unbiased high-throughput functional screening techniques, assessing migration and morphology, and combine them with transcriptomic analysis of prostate cancer cell lines and *in silico* analysis of patient datasets.

As it is technically challenging to study cellular migration *in vivo*, particularly in a high-throughput fashion, several *in vitro* models have been established to mimic this (5, 6). Among these, the “wound healing” or “scratch” assay is the most commonly used technique (7), owing to the simplicity and low cost of its set-up. There have been previous reports in which the scratch assay was scaled up to 96- or 384-well plates, for use in high-throughput screening for migration (8), using pin tools attached to robots (9). Alternative approaches have also been reported, including the use of exclusion zone technology to create cell-free regions for subsequent analysis of cell movement (10). It has been reported that a spindle-like morphology is associated with an epithelial-mesenchymal transition (EMT) gene signature (11), and that a change in morphology, due to alterations in cell-cell adhesion interactions and cellular protrusions, is an important parameter associated with directed cell migration *in vitro* (12, 13). Here we employ a 96-pin scratch tool for the migration screen, and concurrently perform high content imaging to analyze morphological changes indicative of epithelial or mesenchymal morphology. Utilizing a microRNA mimic library, we have identified a number of microRNAs that control both migration and morphological changes. Transcriptomic analysis, and integration of functional and expression data with analysis of clinical datasets have enabled the identification of microRNAs and a microRNA seed sequence that are strongly linked to metastatic behavior and prostate cancer progression.

## Materials and Methods

### Cell Culture

PC3-EGFP cells were a gift from Yolanda Calle (Kings College London), and were cultured in RPMI 1640 medium with L-glutamine, sodium pyruvate, MEM non-essential amino acids, MEM vitamins, 10% fetal bovine serum, and penicillin-streptomycin. ARCaPE and ARCaPM cells were purchased from Novicure, Inc., USA, and were cultured in MCaP medium with 5% fetal bovine serum and penicillin-streptomycin as described previously (14).

### MicroRNA Mimic Library

A human microRNA mimic library from Dharmacon (CS-001010 Human Mimics Lot 10100, CS-001015 Supplement Human Mimic 16.0 Lot 11144), corresponding to Mirbase version 16.0 was used for this study.

### Transfection and Cell Seeding for High-Throughput Screens

Lipofectamine RNAiMax reagent was used for transfection, according to the manufacturer's recommendations. Briefly, RNAiMax reagent was diluted in Opti-MEM and mixed with microRNA mimics, and was aliquoted manually into tissue culture-treated 96-well plates (Perkin-Elmer). Cells were then seeded into these wells using an automated liquid handling system at 20,000 cells per well, resulting in a final concentration of 25 nM of the microRNA mimic or controls. For the morphology screen, cells were seeded at a density of 7,500 cells per well, and transfected as above.

### Scratch Assay

Twenty-four hours post-transfection, confluent monolayers of cells were scratched uniformly using a 96-pin scratch tool called WoundMaker (IncuCyte® Cell Migration Kit, Cat No 4493, Essen Bioscience), and washed twice with phosphate buffered saline using the automated liquid handling system to remove floating cells. The wells were then replaced with cell culture medium.

### High-Content Imaging

All high-content imaging was performed using the InCell Analyser 6000 Cell Imaging System (GE Healthcare Life Sciences). Images for the migration screen were obtained at 0 h (i.e., immediately after the scratch was performed), 6, 12, 18, and 24 h, at 4X magnification in both bright-field and green fluorescent channels. For the morphology screen, images were obtained 24 and 48 h post-transfection in the green fluorescent channel at 10X magnification.

### Migration Analysis

The area of the scratch was extracted using the InCell Analysis software, for each well and for each time point. The area from 0 h was subtracted from that of all subsequent time points to yield the migration of the cells in the corresponding duration. Data from non-targeting control-transfected wells (negative controls) were used for per-plate normalization, to reduce plate and batch-effects (Supplementary Figure 2), using the CellHTS2 package (15) (version 2.40.0) in R/Bioconductor.

### Morphology Analysis

The images were segmented and cell outlines (“objects”) extracted using CellProfiler software (16). These objects were further filtered based on size to eliminate cell debris and imaging artifacts. Following this, CellProfiler was used to extract features describing the shape of the objects. Eccentricity was selected for single feature analysis, using the CellHTS2 package. As above, negative controls were used for per-plate normalization (Supplementary Figure 7). For multi-feature analysis, data from all control wells (~3,000 wells) were divided equally into a training set and test set. For the training set, non-targeting controls and mock-transfected wells were classified as mesenchymal, miR-373 wells as epithelial (based on a visible change to epithelial morphology and it coming up as a candidate in single feature analysis based on Eccentricity alone), and siPTK6 and miCon-transfected wells as intermediate morphologies. From the training set, secondary features (i.e., features derived from some combination of primary features like radius, diameter, major axis length, etc.) were used to build a linear discriminant analysis model. The secondary features used were Area, Compactness, Eccentricity, EulerNumber, Extent, FormFactor, and Solidity. The linear discriminant model was then applied on the test set to determine the accuracy of the model. Finally, the model was applied to the unknown samples to classify them into epithelial, intermediate, and mesenchymal morphologies.

### Identification of Hits

Z-scores were calculated for the normalized migration and morphology data using the CellHTS2 package. A Z-score cut-off of −1 was used for microRNAs that inhibit migration, and a cut-off of +1 for those that promote migration. Similarly, Z-score cut-offs of −1 and +1 were used for rounded and spindle shapes in the morphology (eccentricity) screen.

### Annotation

The microRNA mimic library, RNA-seq data, and microRNA expression data from public datasets used different formats for microRNA annotation. Hence, these disparate formats were reconciled using the microRNA sequences, and were matched to the latest Mirbase (version 21) nomenclature (17, 18). All microRNA names in this study are referred to in this format.

### MicroRNA Sequencing and Analysis

Total RNA was extracted from near-confluent ARCaPE and ARCaPM cells in triplicates, size-selected for small RNAs (<200 bases), adapter-ligated and sequenced on the Illumina HiSeq2000 platform, at the Wellcome Trust Centre for Human Genetics, University of Oxford. Sequencing data thus obtained were checked for quality and correlation between replicates (Supplementary Figure 11) and microRNA counts were obtained using Chimira (version 1.0) (19). Differential expression analysis was performed using DESeq2 (20).

### Data Mining

MicroRNA expression and clinical data in the Taylor dataset (GSE21036) were downloaded from cBioportal and analyzed in R statistical software (21). Viability data for the PC3 cell line were downloaded from the Lethal MicroRNA Database (http://microrna.garvan.unsw.edu.au/mtp/database/index).

### Target Analysis

Experimentally validated targets were downloaded from miRTarBase Release 7.0 (22). The database was filtered for microRNAs of clinical interest and the target genes were sorted by the number of microRNAs targeting them.

### Seed Analysis

A 6-nucleotide sequence in the positions 2–7 from the 5′ end was considered as the seed sequence.

### Network Analysis

All microRNA: known target interactions were downloaded directly from DIANA Tarbase with approval (23). A subset of the data containing four microRNAs was used for this analysis.

### Statistical Analysis

All statistical analyses were performed in R (version 3.4) and Bioconductor (version 3.5). Correlation analyses were performed using Pearson method. For survival analyses, Cox Proportional Hazard (univariate) model was used, with Bonferroni correction for multiple testing. Over-representation of the seed sequence was analyzed using Fisher's exact test. Student's *t*-test (unpaired, two-tailed) was used for comparison of two groups (qRT-PCR). For all statistical analysis, the significance level, α, was set at 0.05. For the phenotypic screening experiments, three technical replicates (cells seeded and microRNA mimics transfected on the same day for all replicates) were used for each microRNA mimic library plate. All plates processed on the same day were defined as a batch. For small RNA sequencing, three technical replicates (RNA extracted on the same day from cells of the same passage number, seeded in 3 wells each) were used for each cell line.

## Results

### Functional Screening for miRNAs Regulating Prostate Cancer Cell Migration

**Figure 1:**
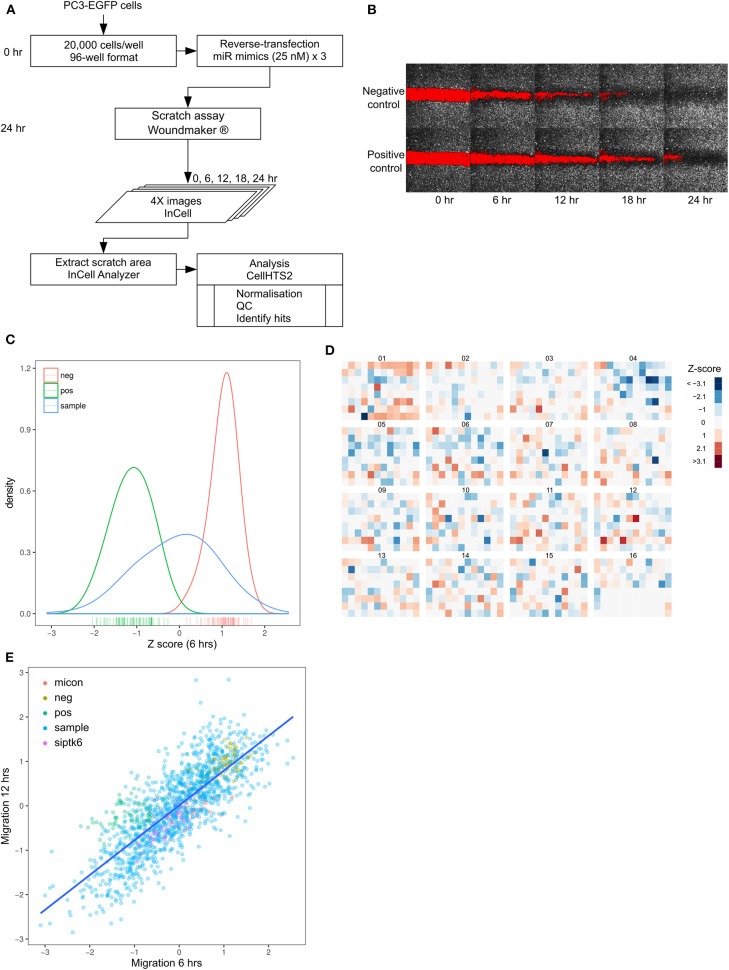
High throughput screening identified miRNAs that regulate prostate cancer migration. **(A)** Workflow of migration screen. **(B)** Images of 96-well plate wells transfected with non-targeting control siRNA (Negative control) or miR-373 (Positive control) at 4X magnification, obtained at 6-hourly intervals. **(C)** Density distribution of negative (red) and positive (green) controls in the entire screen, compared with that of the mimic library (blue). **(D)** Heatmap of normalized and averaged migration z-scores in the entire library. **(E)** Relationship between migration Z-scores from the first (6 h) and second (12 h) time-points across the entire library.

Increased migration is a key characteristic of metastasis. In order to systematically identify microRNAs that regulate migration in prostate cancer cells, we chose a 2D migration model, commonly known as a wound healing assay or scratch assay, which was scalable and cost-effective to develop to a large high-throughput screen. We performed a high-content, fluorescence-based, high-throughput screening in PC3-EGFP prostate cancer cells using a library of 1,253 microRNAs (Mirbase version 16). Scratches were uniformly generated in PC3-EGFP cells using a WoundMaker, following transfection with the library of miRNA mimics and image analysis performed to quantify migration (Figure 1A, Supplementary Figure 1). From the microscopy images, extent of migration was represented as the area of the gap closed at each time point, relative to time = 0 (Figure 1B). Q-Q plots of per-plate normalized (Supplementary Figure 2) migration data for each time point demonstrated an overall Gaussian distribution (Supplementary Figure 3). Non-targeting siRNA- and mock-transfected cells were used as negative controls, and miR-373-transfected cells were used as a positive control (24). There was limited overlap between the kernel density estimate curves of negative and positive controls (Figure 1C) and the mean normalized Z-score was −1.11 for the positive controls, and +1.04 for the negative controls. The screening was performed in triplicate and the replicates showed good reproducibility, seen by a pairwise correlation of >0.85 among all replicates (Supplementary Figure 4). No edge effects were detected in any library plates, with the distinct pattern in plate 1 reflecting the distribution of a specific set of miRNAs in this plate (Figure 1D). A Z-score cut-off of −1 and +1 was used to identify “hits,” i.e., microRNAs that inhibit migration and promote migration, respectively (Figure 1D), and a strong positive correlation (Pearson's *r* = 0.80, *p* < 0.001) was found between time points across the entire library (Figure 1E). The screening identified 239 miRNAs with a Z-score <-1 and 192 miRNAs with a Z-score > +1 (Figure 1D). Z-score cut-offs were designed to be less stringent than those commonly applied, since PC3-EGFP cells are highly migratory and subsequent analysis was only performed with those microRNAs that inhibit migration (i.e., only limited to the left of the distribution curve).

**Figure 2:**
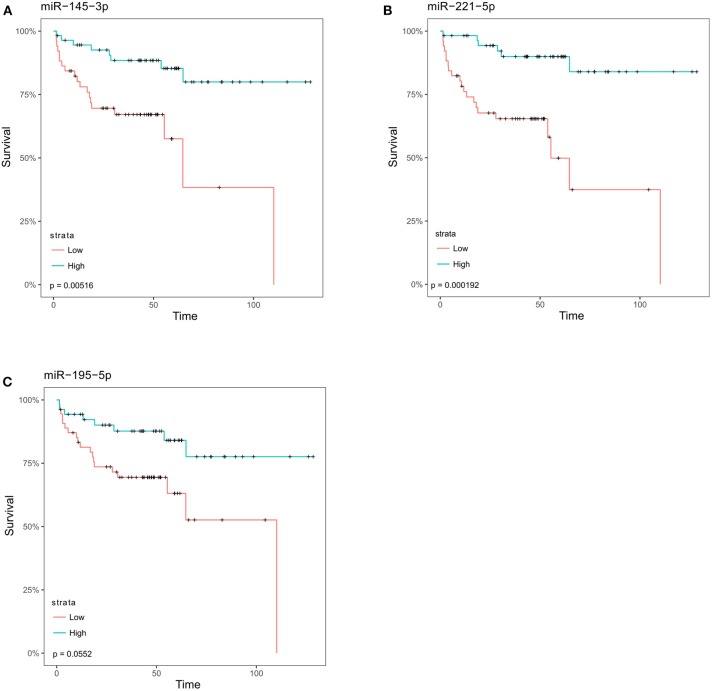
MicroRNAs identified as inhibitory in migration screen are associated with an increase in disease-free survival. Following analysis of the Taylor dataset, those microRNAs that were significantly decreased in metastatic prostate cancer as compared to primary tumor were overlapped with microRNAs found to inhibit migration (Z-score < −1). The overlapping samples were then stratified into low- and high-expressing groups relative to the median for each microRNA. Kaplan Meier survival curves for **(A)** miR-145-3p, **(B)** miR-221-5p, **(C)** miR-195-5p. *p*-values shown are corrected for multiple testing using the Bonferroni method.

To determine whether these microRNAs were also detectable in clinical samples and associated with advanced disease we performed *in silico* differential expression analysis of the Taylor dataset (25). This revealed 55 microRNAs that were significantly down-regulated (log FC > 1) in metastatic prostate cancer samples compared to primary tumor tissue (Supplementary Table 1). When these microRNAs were overlapped with microRNAs that inhibit migration in the screen, six microRNAs (miR-145-3p, -145-5p, 195-5p, 221-3p, -221-5p, 222-3p) were found to be common between the two datasets (Table 1, Supplementary Figure 5). Survival analysis of these microRNAs in the Taylor dataset demonstrated that low expression of miR-145-3p, miR-221-5p or miR-195-5p resulted in significantly worse survival (Figure 2; Bonferroni corrected *p*-value ≤ 0.05).

**Table 1:** MicroRNAs identified as inhibitory in migration screen and with decreased expression in metastatic prostate cancer samples.

**MicroRNA**	**Log2FC**	**Diff. exp. adj *p*-value**	**Survival HR**	**Survival adj. *p*-value**
hsa-miR-145-3p	3.34	4.03E-27	3.934	0.00516
hsa-miR-145-5p	3.22	8.00E-25	1.681	1
hsa-miR-195-5p	1.31	1.66E-05	2.898	0.0522
hsa-miR-221-3p	2.41	6.62E-16	2.407	0.156
hsa-miR-221-5p	1.62	6.96E-08	5.8	0.000192
hsa-miR-222-3p	1.87	1.38E-08	1.049	1

*MicroRNAs inhibiting migration (Z-score < −1)*.

*MicroRNAs differentially expressed between in primary and metastatic prostate cancer samples in the Taylor dataset (log2 Fold Change, log2FC > 1)*.

*Survival—hazard ratios (HR) and corresponding Bonferroni-adjusted p-values for differences in survival between low- and high-expressing patients (univariate analysis, Cox Proportional Hazard model)*.

### Functional Screening for microRNAs Regulating Morphology

**Figure 3:**
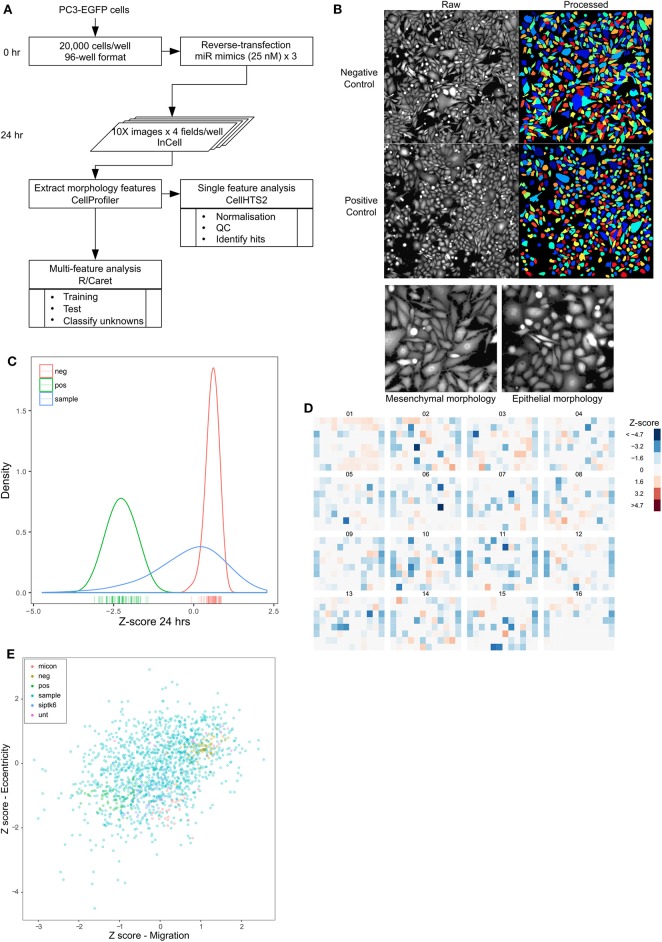
High content screening identifies miRNAs that alter prostate cancer cell morphology. **(A)** Workflow of morphology screen. **(B)** Images of 96-well plate wells transfected with non-targeting control siRNA (Negative control) or miR-373 (Positive control) at 10X magnification, obtained 24 h after transfection (left half), and segmented objects identified using CellProfiler (right half). Colors represent individual cells to indicate segmentation. **(C)** Density distribution of negative (green) and positive (red) controls in the entire screen, compared with that of the mimic library (blue). **(D)** Heatmap of normalized and averaged morphology z-scores in the entire library. **(E)** Z-scores for migration and eccentricity data for each microRNA, showing a significant moderate correlation (controls alone—Pearson's *r* = 0.8, *p* < 0.001, all samples—Pearson's *r* = 0.36, *p* < 0.001), respectively, between the two.

Epithelial-to-mesenchymal transition is considered to be a key component of metastatic progression. *In vitro*, the epithelial phenotype is characterized by a rounded morphology, whereas, mesenchymal cells tend to be spindle-shaped, a morphology change thought to promote invasion and migration. The shift between epithelial and mesenchymal states is increasingly being recognized as a dynamic process in cancer progression, and this plasticity could be regulated by microRNAs. We developed a second high-throughput screen to characterize the change in shape induced by overexpression of microRNAs as an indicator of epithelial-mesenchymal plasticity (Figure 3A). PC3-EGFP cells were used for this screen, allowing for the use of GFP fluorescence in the morphological analysis. PC3 cells were transfected with a microRNA mimic library as described previously, and microscopy images were acquired 24 h following transfection. The microscopy images were segmented to identify individual cells as objects, with each cell represented by a distinct color so as to distinguish adjacent objects, and morphology features were extracted from these objects (Figure 3B; Supplementary Figure 6). Eccentricity was chosen as a measure of mesenchymal morphology, from among a list of morphological parameters, due to its effectiveness in separating the positive and negative controls (Supplementary Figure 6). Per-plate normalization was performed to account for plate-to-plate variation (Supplementary Figure 7). There was very high concordance between replicates (Pearson's *r* > 0.95) (Supplementary Figure 8) and a Q-Q plot of the data shows a left-skewed normal distribution (Supplementary Figure 9). Kernel density estimate curves of positive (mean Z-score ~ −2) and negative (mean Z-score ~ 0.7) controls were again well-separated with little overlap (Figure 3C). PC3 cells have a spindle-shaped mesenchymal-like morphology and the screening identified 243 miRNAs with a Z-score < -1, indicative of an epithelial transformation and 142 miRNAs with a Z-score >1 that induced a further elongated morphology indicative of a mesenchymal phenotype (Figure 3D). However, similar to the migration screen, only miRs that induce a rounded morphology were analyzed further, as PC3-EGFP cells originally have a mesenchymal morphology. Data from the migration and morphology screens were combined to determine the degree of correlation between the two. A significant correlation was observed between migration and eccentricity for the controls alone (Pearson's *r* = 0.8, *p* < 0.001), and for all samples (Pearson's *r* = 0.36, *p* < 0.001), respectively (Figure 3E), with 94 miRNAs found to both inhibit migration and induce a rounded morphology indicative of driving an epithelial phenotype (Figure 3E).

As for the migration data, microRNAs downregulated in metastatic samples in the MSKCC dataset were overlapped with microRNAs that induce a rounded morphology, identifying six microRNAs (hsa-let-7e-5p, hsa-miR-101-3p, hsa-miR-130a-3p, hsa-miR-148a-3p, hsa-miR-214-3p, hsa-miR-221-5p) as common between the two datasets (Table 2).

**Table 2:** MicroRNAs that induce a rounded morphology and with reduced expression in metastatic samples.

**MicroRNA**	**Log2FC**	**Diff. Exp. Adj *p*-value**	**Eccentricity score**	**Survival HR**	**Survival adj. *p*-value**
hsa-let-7e-5p	1.29	6.06E-05	−1.13	2.95	0.0432
hsa-miR-101-3p	1.07	1.25E-08	−1.75	2.70	0.078
hsa-miR-130a-3p	1.96	2.42E-17	−1.52	2.31	0.198
hsa-miR-148a-3p	1.45	1.07E-06	−2.27	1.60	1
hsa-miR-214-3p	1.11	1.51E-07	−1.75	1.02	1
hsa-miR-221-5p	1.62	6.96E-08	−1.62	5.80	0.000192

*MicroRNAs differentially expressed between in primary and metastatic prostate cancer samples in the Taylor dataset (log2 Fold Change, log2FC > 1)*.

*MicroRNAs inducing rounded morphology (eccentricity Z-score < −1)*.

*Survival—hazard ratios (HR) and corresponding Bonferroni-adjusted p-values for differences in survival between low- and high-expressing patients (univariate analysis, Cox Proportional Hazard model)*.

To confirm that analysis of morphology using eccentricity alone is robust, we performed a multi-feature analysis with several morphology measures. A linear discriminant analysis model was trained with half of the control samples (training set) to classify them into epithelial, intermediate, or mesenchymal phenotypes (Supplementary Figure 10A). The model was then applied to a test set to calculate the mis-classification rate (Supplementary Figure 10B), and finally applied to unknown samples. This resulted in a much smaller set of samples classified as “epithelial” morphology, all of which, except one, were also identified as candidates using single feature analysis (Eccentricity Z-score < −1) (Supplementary Figures 10C,D).

### Cell Viability Is an Important Confounder for Migration

**Figure 4:**
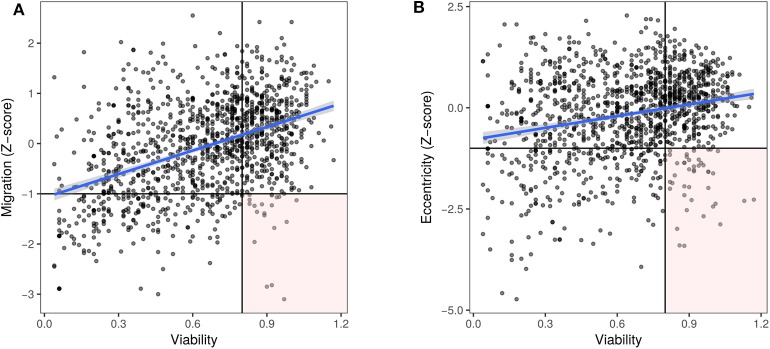
Decreased viability is associated with decreased migration. Viability data for PC3 cells (26), represented as value normalized to controls was plotted along the x axis and for the corresponding microRNAs, **(A)** migration (Pearson's *r* = 0.43, *p* < 0.001) or **(B)** eccentricity (Pearson's *r* = 0.23, *p* < 0.001) Z-scores were plotted along the y axis, respectively. MicroRNAs in the lower right quadrant (Z-score < −1, viability > 0.8) were considered to inhibit migration and induce a rounded morphology independent of cell death.

Decreased cell viability caused by specific microRNAs (either by decreased proliferation or increased cell death) can result in apparently decreased migration as measured by the scratch assay. Further, dying cells may detach from the culture surface, appearing as rounded cells. Hence, we chose to consider the effect of microRNAs on the viability of prostate cancer cells, to identify microRNAs that strictly reduce only migration or eccentricity. PC3 cell viability data from Nikolic et al. (26) were used to account for any confounding of migration and morphology data. A moderate positive correlation (Pearson's *r* = 0.43, *p* < 0.001) was noted between migration and cell viability (Figure 4A). On the other hand, there was a mild positive correlation (*r* = 0.23, *p* < 0.001) between eccentricity and cell viability (Figure 4B). In both cases, a lower viability cut-off of 0.8 and an upper cut-off of 1.2 were used to distinguish microRNAs that primarily affect migration or change in morphology without affecting cell viability (Figures 4A,B, Supplementary Tables 2, 3).

### Transcriptomic Analysis Reveals Distinct miRNA Profiles Associated With EMT in Prostate Cancer

**Figure 5:**
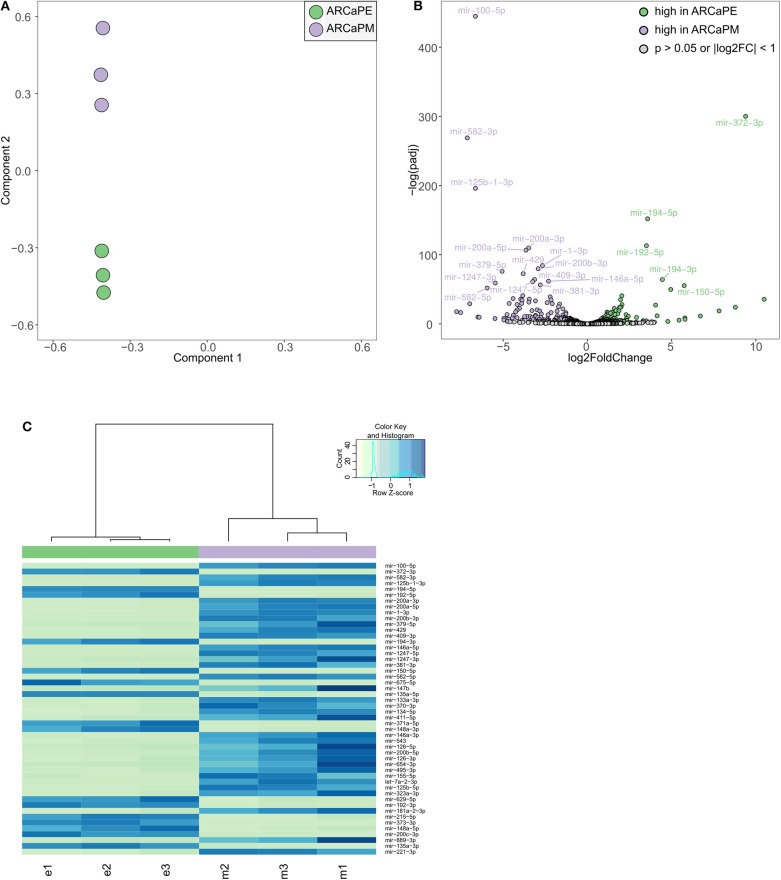
Differential microRNA expression in epithelial and mesenchymal prostate cancer cells. **(A)** Principal component analysis of small RNA sequencing data from ARCaPE and ARCaPM human prostate cancer cell lines, in triplicates. **(B)** Volcano plot of significantly differentially expressed microRNAs (*p* < 0.05, log2 fold change > 1). **(C)** Heatmap of the top 50 differentially expressed microRNAs (e = ARCaPE, m = ARCaPM).

Taken together, our screening strategies have identified a number of microRNAs that have functional effects to dysregulate migration and/or morphology. To further interrogate the role of microRNAs in prostate cancer metastasis, we have undertaken transcriptomic analysis in a cellular model of epithelial-mesenchymal transition. ARCaPE and ARCaPM human prostate cancer cell lines, derived from the parental line ARCaP, have been well-established as a model of epithelial-mesenchymal transition in prostate cancer (27–31). Importantly, they show remarkable differences in their ability to metastasise to bone and other organs *in vivo*, aligned with distinct phenotypic differences *in vitro*. Hence, we sought to identify microRNAs that are differentially expressed between these two cell lines, which may contribute to their functional metastatic differences. Small RNA (<200 bases) from both these cell lines was subjected to sequencing in triplicates (Supplementary Figure 11). Principal component analysis confirmed a distinct clustering of the two cell lines (Figure 5A). Unsupervised hierarchical clustering confirmed that microRNA expression profiles are distinctly different between the two cell lines, with 119 miRNAs significantly increased and 81 microRNAs significantly decreased in ARCaPM cells as compared to ARCaPE (Figures 5B,C, Supplementary Table 4). Further analysis revealed that many of the differentially expressed microRNAs belong to only a few microRNA families/clusters. Unsurprisingly, microRNAs in these clusters are co-expressed due to common promoters, which are known to be regulated by Wnt signaling (32) and BMP signaling (33) pathways. The microRNAs that were most strikingly overexpressed in ARCaPE cells belong to the miR-372 and miR-302 clusters, which have been previously shown to be key regulators of EMT in embryonic stem cells (34) and induced pluripotent stem cells (35).

### Integration of Functional Screening With Transcriptomic Profiling Identifies miRNAs and Seed Sequences Associated With Prostate Cancer Metastasis

**Figure 6:**
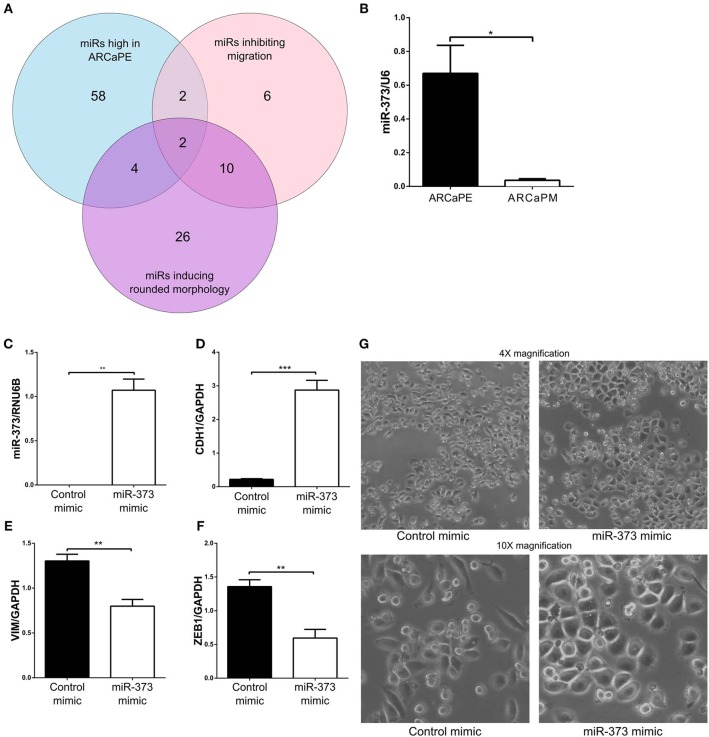
Integration of functional microRNA screening and differential microRNA expression. **(A)** MicroRNAs that were significantly highly expressed in ARCaPE prostate cancer cells (“miRs high in ARCaPE”) were intersected with hits from the migration screen (“miRs inhibiting migration,” Z-score < −1, viability > 0.8) and hits from the morphology screen (“miRs inducing rounded morphology,” Eccentricity Z-score < −1, viability > 0.8) to identify two microRNAs that were common among the three datasets. **(B)** MiR-373-3p expression in ARCaPE and ARCaPM cell lines, measured by real-time PCR. **(C)** Confirmation of MiR-373-3p overexpression in ARCaPM cells by reverse-transfection of the mimic. Total RNA was extracted from the miR-373-3p overexpressed cells 48 h post-transfection, and the corresponding cDNA was analyzed for E-cadherin **(D)**, vimentin **(E)**, and ZEB1 **(F)** mRNA expression. **(G)** Change in morphology was detected by phase-contrast microscopy at 4X and 10X magnification (*p < 0.05, **p < 0.01, ***p < 0.001, unpaired Student's *t*-test).

When “miRs inhibiting migration” (migration Z-score < −1, viability > 0.8) and “miRs inducing rounded morphology,” (Eccentricity Z-score < −1, viability > 0.8) were combined with differentially expressed miRs from the RNA-seq data, there was minimal overlap between all the datasets, with only two microRNAs miR-373-3p and miR-302d-3p found to inhibit migration, induce a rounded morphology and exhibit higher expression in the epithelial ARCaPE cell line (Figure 6A, Table 3). MiR-373 was confirmed to be expressed at higher levels in ARCaPE cells compared to ARCaPM cells (Figure 6B). Overexpression of miR-373 in ARCaPM cells (Figure 6C) resulted in significantly increased E-cadherin, and significantly decreased vimentin and ZEB1 mRNA expression (Figures 6D–F, *p* < 0.01). There is also a distinct shift in miR-373-overexpressing ARCaPM cells from mesenchymal to epithelial morphology (Figure 6G), supportive of epithelial plasticity.

**Table 3:** MicroRNAs common among all three datasets (inhibiting migration, inducing rounded morphology, elevated expression in ARCaPE cells).

**Mirbase name**	**Mature sequence**	**seed**	**Migration score**	**Eccentricity score**	**Log2FC**
hsa-mir-302d-3p	UAAGUGCUUCCAUGUUUGAGUGU	AAGUGC	−1.06	−1.36	1.36
hsa-mir-373-3p	GAAGUGCUUCGAUUUUGGGGUGU	AAGUGC	−1.56	−1.98	8.78

*Seed sequence—position 2–7*.

*Migration Z-score < −1 and viability > 0.8*.

*Eccentricity (morphology) Z-score < −1 and viability > 0.8*.

*Log2FC (Fold Change) (ARCaPE vs. ARCaPM microRNA expression) > 1*.

**Figure 7:**
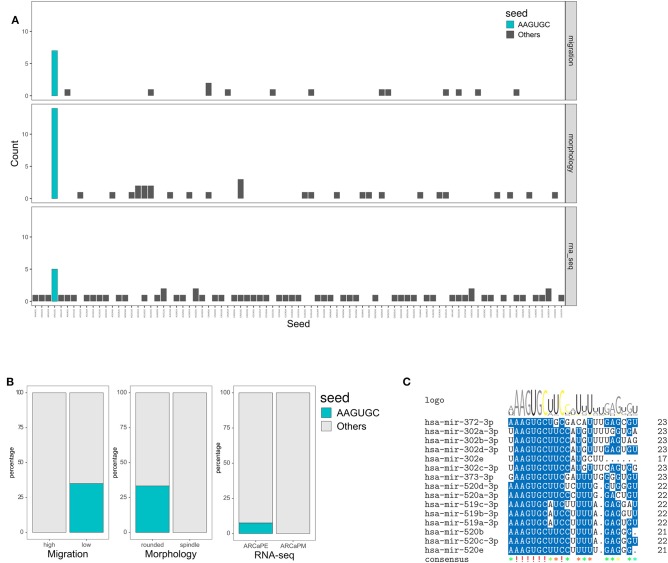
Overrepresentation of AAGUGC seed sequence associated with integrated screening and transcriptomic analysis. **(A)** Number of occurrences of each seed sequence in miRs that inhibit migration but do not decrease viability (Z score < −1, viability > 0.8), induce rounded morphology but do not decrease viability (Z-score < −1, viability > 0.8) and miRs expressed highly in ARCaPE cells (log2FC > 1). **(B)** Over-representation of the AAGUGC seed sequence was tested in the migration (high vs. low), morphology (rounded vs. spindle shapes) and RNA-seq (ARCaPE vs. ARCaPM cell lines) datasets. *P* < 0.01, Fisher's Exact test. **(C)** Sequence homology among MicroRNAs sharing the AAGUGC seed sequence.

Further investigation revealed that the AAGUGC seed sequence was overrepresented (*p* < 0.01) in all three datasets (high in ARCaPE, inhibiting migration, and inducing rounded morphology, but not affecting viability), suggesting a common functional role of this sequence in regulating migration, morphology and EMT (Figures 7A,B). In support, seed analysis of samples classified as “epithelial” by the linear discrimination analysis model revealed an over-representation of the AAGUGC sequence (Supplementary Figure 10D). MicroRNAs that share this seed sequence belong to three main families, miR-372, miR-302 and miR-520 (Figure 7C). In addition to the seed sequence, a homology can be noted in the positions 10, 13, 15, 20 for bases C, U, U, and G, respectively. On the other hand, microRNAs with the AAGUGC motif in positions 3–8 (belonging predominantly to microRNAs of the miR-17-92 cluster) have additional homology in positions 2, 15, 18, and 19 for A, U, A, and G, respectively (Supplementary Figure 12A). The mean Z-scores of miRs with AAGUGC at position 2–7 were −1.03 (migration) and −2.05 (morphology), as opposed to those with AAGUGC at position 3–8, which were 0.23 (migration) and −0.05 (morphology) (Supplementary Figure 12B). All microRNAs with the AAGUGC motif anywhere in their sequence is also shown for comparison (Supplementary Figure 12C).

## Discussion

Once prostate cancer has metastasized, the tumor becomes refractory to current treatment approaches and the malignancy is largely incurable. Understanding the molecular control of the metastatic process is critical in order to develop new effective approaches to combat this advanced disease. MicroRNAs cause changes in phenotype through the regulation of a network of target messenger RNAs. While there has been much focus on direct targets, functional characterization of the microRNAs deserves more attention, and is arguably more relevant to study cancer cell behavior. Phenotypic screens have previously been used to identify key microRNA regulators of cell function, but here we developed a novel, integrative screening approach designed to identify key molecular regulators of metastatic progression by combining multiple functional analyses. As evidence is accumulating for the importance of microRNAs in prostate cancer, we used our integrative screening to identify key microRNAs that were associated with prostate cancer migration and EMT. The overall aim of this study was to use the functional screen to identify candidates that are of relevance in the clinical context, as well as in the context of epithelial-mesenchymal plasticity (in which migration and morphology are key functional readouts).

One of the most important aspect of the metastatic cascade is the ability of tumor cells to migrate. We have developed a high-throughput migration screen, measuring the movement of cells into the space made by a uniform scratch through a confluent cell layer. The migration screen demonstrated good reproducibility, plate uniformity and statistical validation metrics and enabled the high-throughput functional study of microRNAs on prostate cancer migration. Compared to previous examples of scratch assays performed in a high-throughput manner (8–10, 36), our assay shows similar or superior uniformity across wells. The very nature of the migration assay dictates that a migratory cell line is required, and PC3 prostate cancer cells are well-characterized for their representation of late-stage prostate cancer, migratory ability and metastatic behavior *in vivo*. However, it should be noted that consequently, we predominantly identified microRNAs that inhibit migration and the assay was less sensitive in identifying microRNAs that promote migration.

Progression from localized prostate cancer to advanced disease is associated with a transition of prostate cancer cells from an epithelial phenotype to a more mesenchymal phenotype. The process of EMT is important to drive both local invasion and metastatic spread. One of the defining features of EMT is a change in morphology, from a rounded, epithelial shape to a more elongated spindle-shaped morphology. This morphology change is well-documented in prostate cancer cells, with a change from rounded to spindle-shaped morphology associated with increased metastatic behavior and vice versa. The marked difference in shape renders this morphological change well-suited for high content, high-throughput screening. In the current study, we have developed such a screen, taking advantage of the spindle-shaped morphology of PC3 prostate cancer cells that can be driven to a more rounded epithelial shape. Using automated microscopy, multi-parameter image processing and visualization tools, we have extracted quantitative data on multiple features associated with mesenchymal morphology, with eccentricity most representative of the morphologic changes. As with the migration screen, the morphology screen demonstrated good reproducibility, plate uniformity and statistical validation metrics. Simpson et al. studied the morphology of migrating cells at the leading edge of the scratch (8), whereas our use of a separate screen enabled a more in-depth analysis of changes in morphology. However, the left-skewed normal distribution of the data suggested that the assay is more sensitive for identifying microRNAs that induce an epithelial morphology compared to those that induce a mesenchymal morphology; this is unsurprising since PC3 cells have a spindle morphology *in vitro*. Correlation analysis of the two screens demonstrated a positive correlation between migration and morphology, providing support for the concept that a more mesenchymal morphology is important in driving migration. Previous high-throughput approaches to study the role of microRNAs in prostate cancer include identification of miRs regulating the expression of the androgen receptor (37, 38), and miRs that regulate proliferation (39). While our screens are target-agnostic, and were not explicitly aimed at looking at the role of the androgen receptor, they are complementary to previous screens, and in combination with them, provide valuable insights into the functional role of microRNAs in prostate cancer.

Combining the morphology and migration functional screens with a microRNA mimic library enabled the high throughput evaluation of the functional effect of microRNAs on these key aspects of metastatic behavior. Sixteen percentage of miRNAs were found to inhibit prostate cancer cell migration and 19% were found to alter morphology, highlighting the importance of microRNAs in regulation of these metastatic processes. A limitation of these functional screens is the necessity for over-expression of miRNAs. To address the question of basal levels of microRNAs driving metastasis, we performed transcriptomic analysis of a pair of prostate cancer cell lines known to differ in their epithelial and mesenchymal morphology, migratory behavior and metastasis *in vivo*, revealing distinct expression profiles in the metastatic mesenchymal ARCaPM cells as compared to the non-metastatic, epithelial ARCaPE cells.

One of the challenges of prostate cancer research is the difficulty in isolating and working with primary cells, and as such, the integrative screen developed takes advantage of well-characterized prostate cancer cell lines. To ensure the clinical relevance of our integrative screen approach, we have aligned our results with those from publicly available datasets of microRNA expression profiles from benign, primary prostate cancer or metastatic prostate cancer. This enables the further focusing of the hits identified from the screens, based upon their potential clinical relevance. Due to the paucity of large microRNA expression studies in men with advanced or metastatic prostate cancer, we were limited to only one dataset to study the clinical significance of selected microRNAs. Using this approach, six microRNAs (hsa-miR-145-3p, hsa-miR-145-5p, hsa-miR-195-5p, hsa-miR-221-3p, hsa-miR-221-5p, hsa-miR-222-3p) were found to be both inhibiting migration and show reduced expression in metastatic prostate cancer (vs. primary tumors, Taylor dataset). Among these, low levels of three microRNAs (miR-145-3p, miR-221-5p, and miR-195-5p) identified in our migration screen, were associated with a reduction in disease-free survival. Further, miR-221-5p also induced a rounded morphology in our screen. The significance of this microRNA is highlighted by a study by Kiener et al., where overexpression of the microRNA was shown to reduce migration, proliferation and colony formation in PC-3M-Pro4luc2 prostate cancer cells *in vitro*, and to inhibit extravasation in a zebrafish model *in vivo* (40). It is also interesting to note that the median fold change values for microRNAs that inhibit migration are higher than those for microRNAs that induce a rounded morphology (2.14 vs. 1.37 respectively) in the Taylor dataset, although the difference falls short of statistical significance (*p-*value = 0.052). This may suggest that migration is more important than morphology in the clinical context. We also analyzed miRTarBase (22), a manually curated database of experimentally validated targets, to identify the top 20 genes commonly targeted by the 11 clinically significant microRNAs (Supplementary Table 5). Interestingly, the microRNA processing genes AGO2 and DICER1 are targets of 7 and 6 of these microRNAs respectively. Other common target genes include oncogenes such as MYC, CDK6, CCND1 and the hormonal receptor gene ESR1.

While our individual screening approaches were successful in identifying multiple microRNAs with differential functional effects and/or expression profiles, the integration of the three screens proved effective in revealing those microRNAs that were common to all screens and therefore may have a greater contribution to the metastatic process. Further, the integration of viability data from Nikolic et al. (26) to the analysis added high stringency to the analysis. A moderate correlation was observed between viability and migration, suggesting that for a number of microRNAs, decreased migration may at least partly be due to decreased cell number. A mild correlation was also noted between viability and cell eccentricity. Hence, for further analysis, only microRNAs that do not alter viability were considered to strictly alter migration or morphology. The combination of those microRNAs with high expression in ARCaPE cells, that inhibited migration and induced a rounded morphology (without reducing viability) identified two microRNAs; miR-373-3p, and miR-302d-3p. Both these microRNAs are known to regulate epithelial-mesenchymal transition as well as stem cell behavior by regulating the TGF-β signaling pathway (35, 41, 42). While the integrative approach we utilized in this study accounted for some known shortcomings (e.g., viability), thus yielding a small number of microRNAs as candidates for further study, hits from the individual screens may also be functionally important in their own right.

miR-373-3p has been previously associated with prostate cancer progression, providing strong support for the power of our integrative screening approach to identify key mediators of the metastatic process. miR-373-3p is known to induce mesenchymal-epithelial transition in prostate cancer cells by inducing the expression of E-cadherin (43) or inhibiting ZEB1 post-transcriptionally (24). In contrast, miR-373-3p has been shown to promote invasion and metastasis in breast and colon cancer cells (44), suggesting a changing role depending on tissue context. Interestingly, miR-373-3p was reported to be elevated in high grade prostate cancer, which is counterintuitive to their functional role as inhibitors of migration (45). The miR-302/367 cluster was recently shown by Guo et al. to be elevated in prostate cancer compared to normal prostate tissue, and shown to promote proliferation and androgen-independence by targeting the tumor suppressor gene LATS2 (46), suggesting that the role of these microRNAs may be a cumulative effect of several functional phenotypes. It should be noted, however, that Guo et al. transfected the entire miR-302/367 cluster into prostate cancer cells, whereas in our study, each microRNA was studied individually highlighting the importance of miR-302d-3p.

MicroRNAs exhibit a sequence specific function, and a 6–8 base region at their 5' end, the seed region, is important to this specificity (47). Analysis of the seed sequences revealed that the AAGUGC was over-represented in all the above three datasets. A shared seed sequence and a further homology in other positions of microRNAs belonging to four microRNA families (miR-372, miR-302, miR-520, and miR-519), may together account for a shared set of targets and consequently, a shared function. Interestingly, Zhou et al. reported that miRs with the AAGUGC motif are oncogenic in non-small cell lung cancer cells, increasing their proliferation (48). While their definition of the AAGUGC motif included miRs with this sequence occurring anywhere in the seed region, we used a stricter definition for the AAGUGC seed, as those sharing the sequence in the 2–7 position appear to have a distinct function in our migration and morphology screens compared to those with this sequence in the 3–8 position. Sinkkonen et al. reported that microRNAs sharing the AAGUGC seed sequence are specific to mouse embryonic stem cells, and regulate DNA methylation in differentiating ES cells (49) and the miR-302 and miR-372 families are well-characterized as regulators of EMT in embryonic stem cells (34, 50). In prostate cancer, in addition to the known role of miR-373 in inducing mesenchymal-epithelial transition, another microRNA miR-371a-3p, which belongs to the same family and contains the AAGUGC sequence at position 1–6, is known to down-regulate the androgen receptor (37).

Taken together, we have developed an integrative screening approach, which combines functional screening with expression profiling and alignment with clinical data in order to narrow down the candidate microRNAs to those of greatest importance in prostate cancer progression. Using this screen, we have identified both novel microRNAs and a microRNA seed sequence that are strongly linked to metastatic behavior and prostate cancer progression. This approach provides the basis for developing new approaches to prevent disease progression, which could include targeting the specific microRNAs identified, or a detailed cellular and molecular investigation into their mechanisms of action. Further, seed analysis provides novel insights into the functional consequences of motifs and their position in the microRNAs. Thus, this new approach to identifying mechanisms that drive prostate cancer metastasis has implications for understanding cancer pathogenesis and the potential to reveal opportunities for developing innovative treatment approaches.

## Data Availability Statement

The datasets generated for this study can be found in the NCBI Gene Expression Omnibus (GSE145078) (51).

## Author Contributions

SR devised and performed experiments, analyzed data, and prepared manuscript. AH, PK, AS, and CY devised and performed experiments. DE contributed to experimental design. FH contributed to experimental design and concept. CE supervised this research, devised experiments, reviewed data, and prepared manuscript. All authors read and approved the final manuscript.

### Conflict of Interest

The authors declare that the research was conducted in the absence of any commercial or financial relationships that could be construed as a potential conflict of interest.
