# Supplementary material for: Transcriptomic and Functional Screens Reveal MicroRNAs That Modulate Prostate Cancer Metastasis
Source: Front Oncol. 2020 Mar 13;10:292. doi: 10.3389/fonc.2020.00292 (PMC7082744; doi:10.3389/fonc.2020.00292)
Supplement: Supplementary file 1 [file Data_Sheet_1.zip › supplementary_tables.pdf]

Supplementary tables

Supplementary table 1. MicroRNAs downregulated in metastatic samples in the Taylor dataset

	ID	ADJ.P.VAL	P.VALUE	T	B	LOGFC	MIRNA_ID
**1**	hsa-miR-1	1.10E-30	2.95E-33	16.99907	65.15229	5.445934	hsa-miR-1
**2**	hsa-miR-133b	9.38E-29	6.43E-31	15.91065	59.81418	4.376786	hsa-miR-133b
**3**	hsa-miR-143	9.38E-29	7.55E-31	15.87872	59.65536	4.041918	hsa-miR-143
**4**	hsa-miR-145*	4.03E-27	4.32E-29	15.07963	55.64098	3.335141	hsa-miR-145*
**5**	hsa-miR-145	8.00E-25	1.07E-26	14.01276	50.16852	3.215684	hsa-miR-145
**6**	hsa-miR-133a	1.97E-21	3.16E-23	12.5035	42.2378	3.012036	hsa-miR-133a
**7**	hsa-miR-143*	2.50E-19	4.69E-21	11.57451	37.27458	2.593085	hsa-miR-143*
**8**	hsa-miR-130a	2.43E-17	5.21E-19	10.70533	32.60115	1.964885	hsa-miR-130a
**9**	hsa-miR-23b	2.76E-16	6.67E-18	10.23546	30.07253	1.94522	hsa-miR-23b
**10**	hsa-miR-221	6.62E-16	1.78E-17	10.05421	29.09812	2.406665	hsa-miR-221
**11**	hsa-miR-100	6.62E-16	1.95E-17	10.03723	29.00685	2.12416	hsa-miR-100
**12**	hsa-miR-125b	8.98E-16	2.89E-17	9.965062	28.61923	1.871256	hsa-miR-125b
**13**	hsa-miR-24-1*	5.24E-15	1.83E-16	9.624307	26.79189	1.740427	hsa-miR-24-1*
**14**	hsa-miR-27b	1.22E-14	4.58E-16	9.453863	25.88025	1.755808	hsa-miR-27b
**15**	hsa-miR-99a	1.40E-12	5.99E-14	8.54401	21.05628	2.232405	hsa-miR-99a
**16**	hsa-miR-199b-5p	2.20E-11	1.00E-12	8.010002	18.27394	2.233036	hsa-miR-199b-5p
**17**	hsa-miR-205	2.59E-11	1.25E-12	7.967331	18.05367	6.544898	hsa-miR-205
**18**	hsa-miR-377	5.14E-11	2.75E-12	7.815776	17.27414	1.367705	hsa-miR-377
**19**	hsa-miR-135a	7.26E-11	4.09E-12	7.739709	16.8846	2.468894	hsa-miR-135a
**20**	hsa-miR-218	7.87E-11	4.64E-12	7.715153	16.7591	1.698359	hsa-miR-218
**21**	hsa-miR-376c	7.91E-10	4.88E-11	7.2567	14.44089	1.350646	hsa-miR-376c
**22**	hsa-miR-376a	1.01E-09	6.52E-11	7.199566	14.15556	1.341662	hsa-miR-376a
**23**	hsa-miR-24	1.12E-09	7.49E-11	7.172005	14.01821	1.000365	hsa-miR-24
**24**	hsa-miR-199b-3p	1.88E-09	1.31E-10	7.061231	13.46826	1.963035	hsa-miR-199b-3p
**25**	hsa-miR-374b	4.45E-09	3.46E-10	6.867187	12.51318	1.429607	hsa-miR-374b
**26**	hsa-miR-28-5p	4.83E-09	3.88E-10	6.843932	12.39945	1.294334	hsa-miR-28-5p
**27**	hsa-miR-101	1.26E-08	1.05E-09	6.643319	11.42529	1.068435	hsa-miR-101
**28**	hsa-miR-222	1.38E-08	1.18E-09	6.618121	11.30383	1.874026	hsa-miR-222
**29**	hsa-miR-204	3.43E-08	3.13E-09	6.41877	10.35036	2.089625	hsa-miR-204
**30**	hsa-miR-136*	3.57E-08	3.35E-09	6.404907	10.28456	1.143504	hsa-miR-136*
**31**	hsa-miR-152	3.67E-08	3.55E-09	6.393051	10.22834	1.014626	hsa-miR-152
**32**	hsa-miR-363	4.45E-08	4.41E-09	6.347666	10.01359	2.068645	hsa-miR-363
**33**	hsa-miR-221*	6.96E-08	7.29E-09	6.243144	9.521843	1.619536	hsa-miR-221*
**34**	hsa-miR-214	1.52E-07	1.79E-08	6.054196	8.643266	1.107509	hsa-miR-214
**35**	hsa-miR-374a	2.16E-07	2.68E-08	5.968375	8.248801	1.529098	hsa-miR-374a
**36**	hsa-miR-30c	2.16E-07	2.78E-08	5.96008	8.210828	1.044526	hsa-miR-30c
**37**	hsa-miR-26b	2.75E-07	3.69E-08	5.8999	7.936197	1.640196	hsa-miR-26b
**38**	hsa-miR-148a	1.07E-06	1.66E-07	5.571936	6.466533	1.447148	hsa-miR-148a
**39**	hsa-miR-199a-5p	1.49E-06	2.35E-07	5.495288	6.129947	1.033407	hsa-miR-199a-5p
**40**	hsa-miR-582-5p	2.78E-06	4.54E-07	5.347728	5.489706	1.686622	hsa-miR-582-5p
**41**	hsa-miR-136	2.99E-06	4.96E-07	5.327524	5.402857	1.000646	hsa-miR-136
**42**	hsa-miR-29b	4.49E-06	8.18E-07	5.213822	4.917829	1.046529	hsa-miR-29b
**43**	hsa-miR-886-3p	5.96E-06	1.14E-06	5.138393	4.599633	1.327171	hsa-miR-886-3p
**44**	hsa-miR-196b	1.02E-05	2.04E-06	5.001567	4.029916	1.148868	hsa-miR-196b

**45**	hsa-miR-195	1.66E-05	3.56E-06	4.87019	3.492234	1.30514	hsa-miR-195
**46**	hsa-miR-495	2.40E-05	5.47E-06	4.767536	3.078677	1.036127	hsa-miR-495
**47**	hsa-let-7e	6.07E-05	1.46E-05	4.526249	2.130216	1.289679	hsa-let-7e
**48**	hsa-miR-203	6.60E-05	1.61E-05	4.50236	2.038165	1.095179	hsa-miR-203
**49**	hsa-miR-455-3p	0.000102	2.72E-05	4.370572	1.536543	1.244216	hsa-miR-455-3p
**50**	hsa-miR-223	0.000377	0.000113	3.996969	0.173754	1.073799	hsa-miR-223
**51**	hsa-let-7f	0.000463	0.000143	3.934115	-0.04656	1.103321	hsa-let-7f
**52**	hsa-miR-31	0.000648	0.000214	3.823071	-0.42923	1.41494	hsa-miR-31
**53**	hsa-miR-146b-5p	0.001087	0.000396	3.648881	-1.0123	1.157313	hsa-miR-146b-5p
**54**	hsa-miR-150	0.005411	0.002495	3.09149	-2.72907	1.053225	hsa-miR-150
**55**	hsa-miR-375	0.009277	0.004427	2.902951	-3.25564	1.066108	hsa-miR-375

Supplementary table 2. MicroRNAs inhibiting migration, but not affecting viability

	ACCNUM	Z-SCORE	MATURE_SEQUENCE	MIRBASE_NAME	SHORTSEED	VIABILITY
1	MIMAT0000617	-1.02	UAAUACUGCCGGGUAAUGAUGGA	hsa-mir-200c-3p	AAUACU	0.83
2	MIMAT0000718	-1.06	UAAGUGCUUCCAUGUUUGAGUGU	hsa-mir-302d-3p	AAGUGC	0.95
3	MIMAT0000726	-1.56	GAAGUGCUUCGAUUUUGGGGUGU	hsa-mir-373-3p	AAGUGC	0.98
4	MIMAT0002830	-1.21	AAGUGCUUCCUUUUAGAGGGUU	hsa-mir-520f-3p	AGUGCU	0.88
5	MIMAT0002834	-2.85	AAAGUGCUUCCCUUUGGACUGU	hsa-mir-520a-3p	AAGUGC	0.9
6	MIMAT0002837	-1.43	AAAGUGCAUCCUUUUAGAGGUU	hsa-mir-519b-3p	AAGUGC	0.89
7	MIMAT0002856	-1.24	AAAGUGCUUCUCUUUGGUGGGU	hsa-mir-520d-3p	AAGUGC	0.85
8	MIMAT0002858	-2.82	ACAAAGUGCUUCCCUUUAGAGUGU	hsa-mir-520g-3p	CAAAGU	0.92
9	MIMAT0002867	-1.23	ACAAAGUGCUUCCCUUUAGAGU	hsa-mir-520h	CAAAGU	0.92
10	MIMAT0002869	-1.36	AAAGUGCAUCCUUUUAGAGUGU	hsa-mir-519a-3p	AAGUGC	0.86
11	MIMAT0003292	-1.2	AUCCCUUGCAGGGGCUGUUGGGU	hsa-mir-623	UCCCUU	0.83
12	MIMAT0003306	-1.39	UGUGCUUGCUCGUCCCGCCCGCA	hsa-mir-636	GUGCUU	0.89
13	MIMAT0004610	-1.12	CUGGUACAGGCCUGGGGGACAG	hsa-mir-150-3p	UGGUAC	0.83
14	MIMAT0004686	-1.96	ACUGUUGCUAAUAUGCAACUCU	hsa-mir-367-5p	CUGUUG	0.85
15	MIMAT0004986	-1.68	CUGACUGUUGCCGUCCUCCAG	hsa-mir-943	UGACUG	0.93
16	MIMAT0005901	-1.02	ACGCCCUUCCCCCCCUUCUUCA	hsa-mir-1249-3p	CGCCCU	0.92
17	MIMAT0005931	-1.13	UAAGUGCUUCCAUGCUU	hsa-mir-302e	AAGUGC	1.02
18	MIMAT0005956	-3.1	CCAGACAGAAUUCUAUGCACUUUC	hsa-mir-1324	CAGACA	0.97
19	MIMAT0013802	-1.03	GGGGCCUGGCGGUGGGCGG	hsa-mir-2861	GGGCCU	0.92
20	MIMAT0015026	-1.07	GGGGAAAGCGAGUAGGGACAUUU	hsa-mir-3153	GGGAAA	0.94

MicroRNAs inhibiting migration (Z-score < -1)
MicroRNAs not affecting viability (0.8 < viability < 1.2)

Supplementary table 3. MicroRNAs inducing a change to rounded morphology, but not affecting viability

	ACCNUM	Z-SCORE	MATURE_SEQUENCE	MIRBASE_NAME	SHORTSEED	VIABIILITY
**1**	MIMAT0000087	-1.94	UGUAAACAUCCUCGACUGGAAG	hsa-mir-30a-5p	GUAAAC	0.88
**2**	MIMAT0000243	-2.27	UCAGUGCACUACAGAACUUUGU	hsa-mir-148a-3p	CAGUGC	1.17
**3**	MIMAT0000261	-1.06	UAUGGCACUGGUAGAAUUCACU	hsa-mir-183-5p	AUGGCA	0.82
**4**	MIMAT0000425	-1.52	CAGUGCAAUGUUAAAAGGGCAU	hsa-mir-130a-3p	AGUGCA	0.9
**5**	MIMAT0000438	-2.3	UCAGUGCAUGACAGAACUUGG	hsa-mir-152-3p	CAGUGC	1.13
**6**	MIMAT0000684	-2.37	UAAGUGCUUCCAUGUUUUGGUGA	hsa-mir-302a-3p	AAGUGC	1.01
**7**	MIMAT0000715	-1.58	UAAGUGCUUCCAUGUUUUAGUAG	hsa-mir-302b-3p	AAGUGC	0.98
**8**	MIMAT0000718	-1.36	UAAGUGCUUCCAUGUUUGAGUGU	hsa-mir-302d-3p	AAGUGC	0.95
**9**	MIMAT0000724	-2.44	AAAGUGCUGCGACAUUUGAGCGU	hsa-mir-372-3p	AAGUGC	1
**10**	MIMAT0000726	-1.98	GAAGUGCUUCGAUUUUGGGGUGU	hsa-mir-373-3p	AAGUGC	0.98
**11**	MIMAT0000759	-1.58	UCAGUGCAUCACAGAACUUUGU	hsa-mir-148b-3p	CAGUGC	0.98
**12**	MIMAT0002823	-1.45	AAGUGCUGUCAUAGCUGAGGUC	hsa-mir-512-3p	AGUGCU	0.92
**13**	MIMAT0002825	-2.16	AAAGUGCUUCCUUUUUGAGGG	hsa-mir-520e	AAGUGC	0.88
**14**	MIMAT0002827	-1.03	GAGUGCCUUCUUUUGGAGCGUU	hsa-mir-515-3p	AGUGCC	0.99
**15**	MIMAT0002830	-3.41	AAGUGCUUCCUUUUAGAGGGUU	hsa-mir-520f-3p	AGUGCU	0.88
**16**	MIMAT0002832	-2.28	AAAGUGCAUCUUUUUAGAGGAU	hsa-mir-519c-3p	AAGUGC	0.91
**17**	MIMAT0002834	-1.96	AAAGUGCUUCCCUUUGGACUGU	hsa-mir-520a-3p	AAGUGC	0.9
**18**	MIMAT0002837	-2.53	AAAGUGCAUCCUUUUAGAGGUU	hsa-mir-519b-3p	AAGUGC	0.89
**19**	MIMAT0002843	-2.88	AAAGUGCUUCCUUUUAGAGGG	hsa-mir-520b	AAGUGC	1.03
**20**	MIMAT0002846	-2.68	AAAGUGCUUCCUUUUAGAGGGU	hsa-mir-520c-3p	AAGUGC	0.96
**21**	MIMAT0002856	-2.99	AAAGUGCUUCUCUUUGGUGGGU	hsa-mir-520d-3p	AAGUGC	0.85
**22**	MIMAT0002858	-1.06	ACAAAGUGCUUCCCUUUAGAGUGU	hsa-mir-520g-3p	CAAAGU	0.92
**23**	MIMAT0002869	-3.4	AAAGUGCAUCCUUUUAGAGUGU	hsa-mir-519a-3p	AAGUGC	0.86
**24**	MIMAT0003266	-1.62	UACGUCAUCGUUGUCAUCGUCA	hsa-mir-598-3p	ACGUCA	1
**25**	MIMAT0003306	-1.15	UGUGCUUGCUCGUCCCGCCCGCA	hsa-mir-636	GUGCUU	0.89
**26**	MIMAT0003880	-1.86	AGGAAGCCCUGGAGGGGCUGGAG	hsa-mir-671-5p	GGAAGC	0.83
**27**	MIMAT0004571	-1.83	CAUCUUACUGGGCAGCAUUGGA	hsa-mir-200b-5p	AUCUUA	0.82
**28**	MIMAT0004686	-1.37	ACUGUUGCUAAUAUGCAACUCU	hsa-mir-367-5p	CUGUUG	0.85
**29**	MIMAT0004805	-1.36	AGUCAUUGGAGGGUUUGAGCAG	hsa-mir-616-3p	GUCAUU	0.88
**30**	MIMAT0004811	-1.57	CAGUGCCUCGGCAGUGCAGCCC	hsa-mir-33b-3p	AGUGCC	0.92
**31**	MIMAT0004928	-1.42	GUGUGCGGAAAUGCUUCUGCUA	hsa-mir-147b	UGUGCG	0.96
**32**	MIMAT0005931	-1.07	UAAGUGCUUCCAUGCUU	hsa-mir-302e	AAGUGC	1.02
**33**	MIMAT0007888	-1.03	UCUGCCCCCUCCGCUGCUGCCA	hsa-mir-1913	CUGCCC	0.98
**34**	MIMAT0013771	-1.16	UUGCUAGUUGCACUCCUCUCUGU	hsa-mir-449c-3p	UGCUAG	0.82
**35**	MIMAT0015026	-1.28	GGGGAAAGCGAGUAGGGACAUUU	hsa-mir-3153	GGGAAA	0.94
**36**	MIMAT0015069	-1.43	UUGGCCAUGGGGCUGCGCGG	hsa-mir-3187-3p	UGGCCA	0.94
**37**	MIMAT0016881	-1.21	CUUGGGGCAUGGAGUCCCA	hsa-mir-4260	UUGGGG	0.89
**38**	MIMAT0016890	-1.36	AGGAAACAGGGACCCA	hsa-mir-4261	GGAAAC	0.88
**39**	MIMAT0018088	-1.67	CAGUGCAAGUGUAGAUGCCGA	hsa-mir-3666	AGUGCA	0.84
**40**	MIMAT0018093	-2	AGAGCUCACAGCUGUCCUUCUCUA	hsa-mir-3670	GAGCUC	0.82
**41**	MIMAT0018182	-1.54	GAGCAAUGUAGGUAGACUGUUU	hsa-mir-3908	AGCAAU	0.92
**42**	MIMAT0018206	-1.29	GAGGCUGAUGUGAGUAGACCACU	hsa-mir-3929	AGGCUG	0.9

MicroRNAs inducing rounded morphology (Z-score < -1)
MicroRNAs not affecting viability (0.8 < viability < 1.2)
