# Supplementary material for: Transcriptomic and Functional Screens Reveal MicroRNAs That Modulate Prostate Cancer Metastasis
Source: Front Oncol. 2020 Mar 13;10:292. doi: 10.3389/fonc.2020.00292 (PMC7082744; doi:10.3389/fonc.2020.00292)
Supplement: Supplementary file 2 [file Image_1.pdf]

Supplementary figures

S1. Plate configuration for migration and morphology screens: MicroRNAs from the mimic library ("sample") and controls (unt - mock transfected, siptk6 - transfected with siRNA targeting PTK6, micon - microRNA non-targeting control, neg - siRNA non-targeting control, pos - miR-373-3p mimic) were transfected in 96-well plates in the following configuration (each block represents 2 wells).

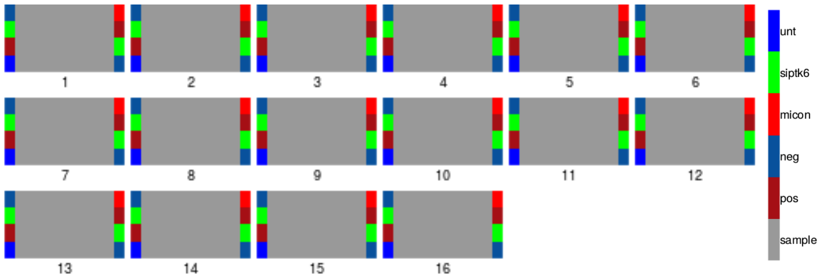

S2. Per-plate normalisation for migration screen (6hrs) : Distribution of migration values across all plates in the screen are shown, before (Raw) and after (Normalised) per-plate normalisation with non-targeting control wells (= negative controls) in CellHTS2.

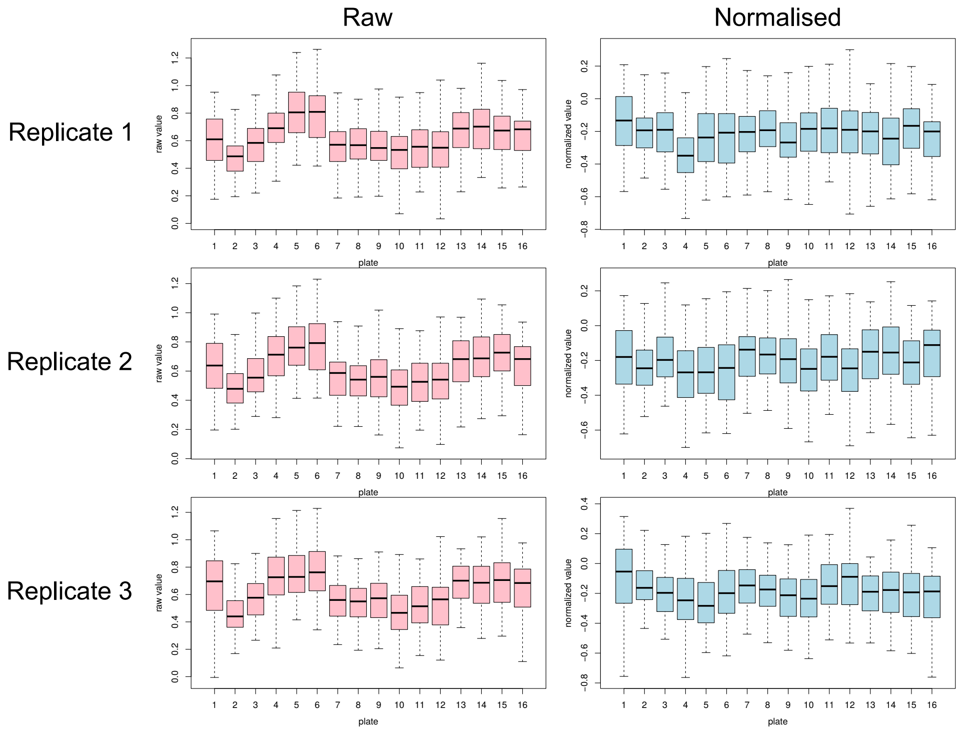

S3. Q-Q plot of migration data: Sample and theoretical quantiles of negative controls (blue), positive controls (red) and samples from the mimic library (black).

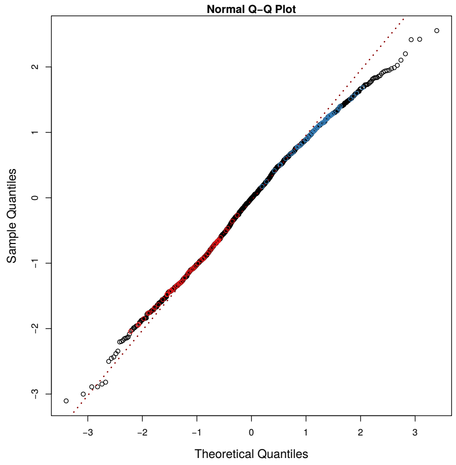

S4. Correlogram: Pair-wise correlation (Pearson r) between replicates in the migration screen, showing high concordance (r > 0.85) between replicates.

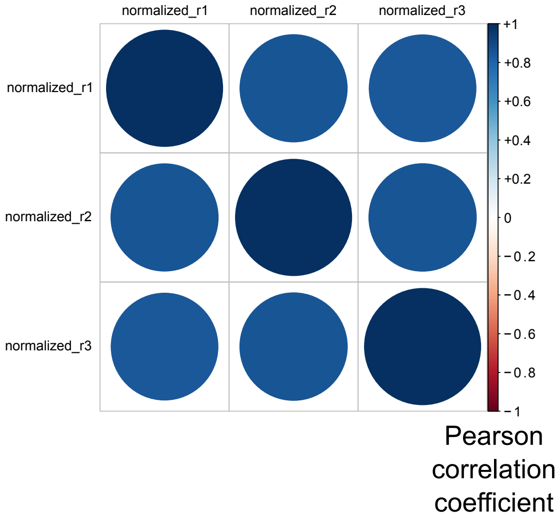

S5. MiR expression in primary and metastatic samples(Taylor dataset): Expression of microRNAs common between low migration and top differentially expressed ones in the Taylor dataset, in metastatic (n = 14) and primary (n = 99) prostate cancer samples.

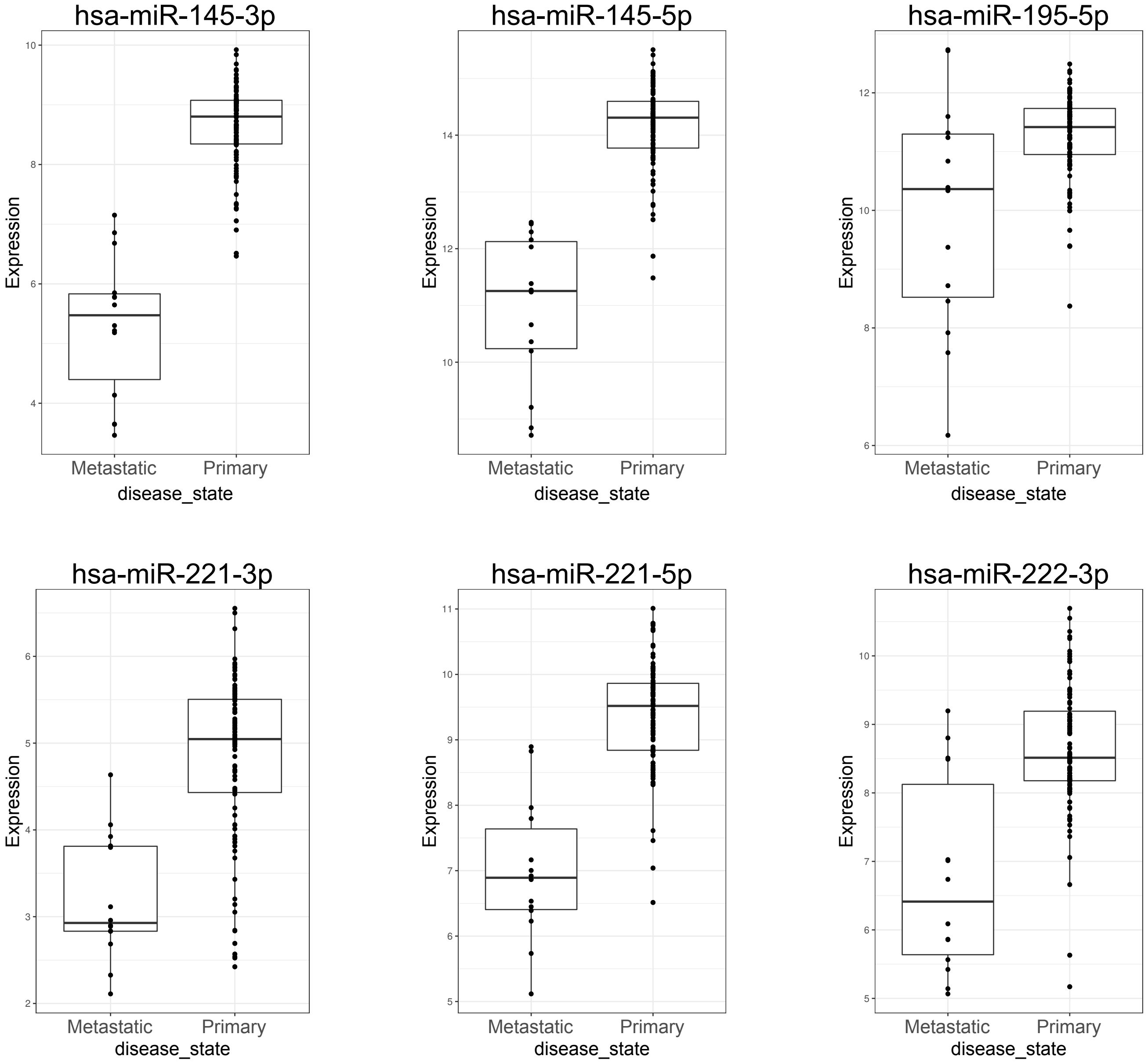

S6. Morphology features: Distribution of morphology features (obtained from CellProfiler analysis) among all screening plates, showing differences in controls. As siCon and mi373 differed the most, they were chosen as negative and positive controls respectively.

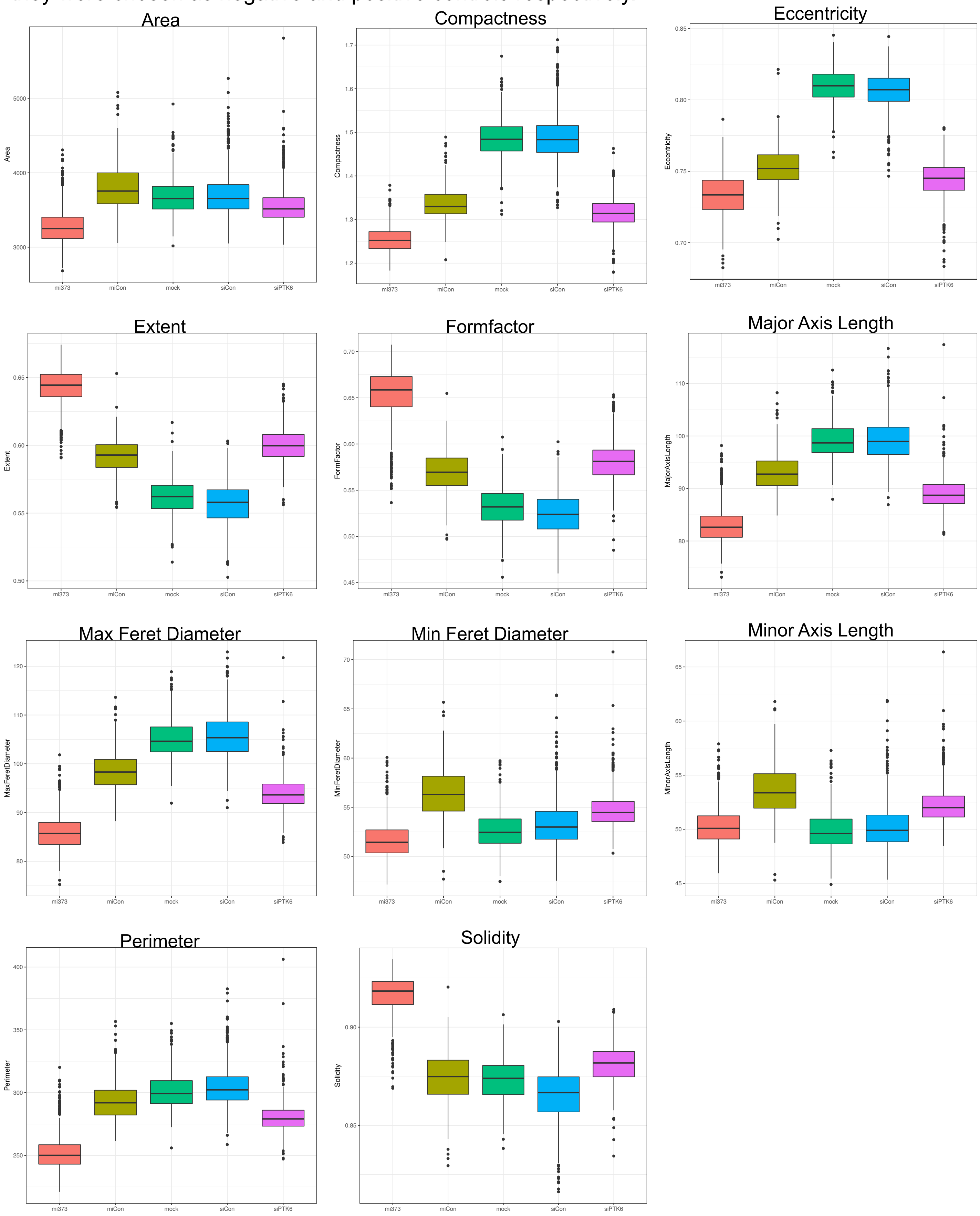

S7. Per-plate normalisation for morphology (24 hrs): Distribution of eccentricity values across all plates in the screen are shown, before (Raw) and after (Normalised) per-plate normalisation with non-targeting control wells (= negative controls) in CellHTS2.

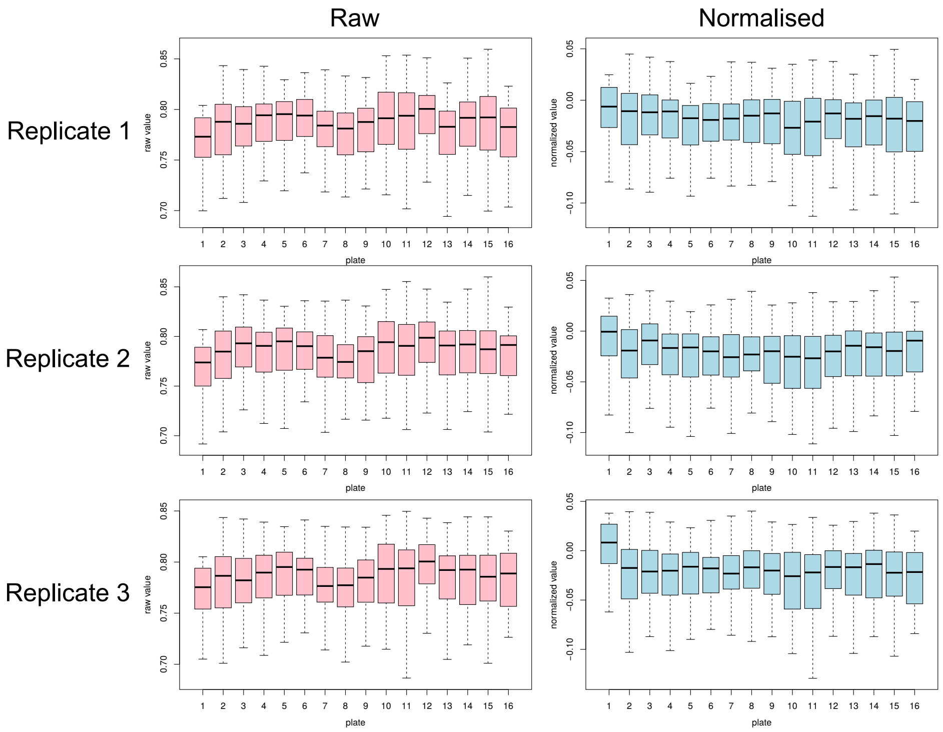

S8. **Correlogram:** Pair-wise correlation (Pearson r) between replicates in the morphology screen, showing high concordance (r > 0.95) between replicates for eccentricity values.

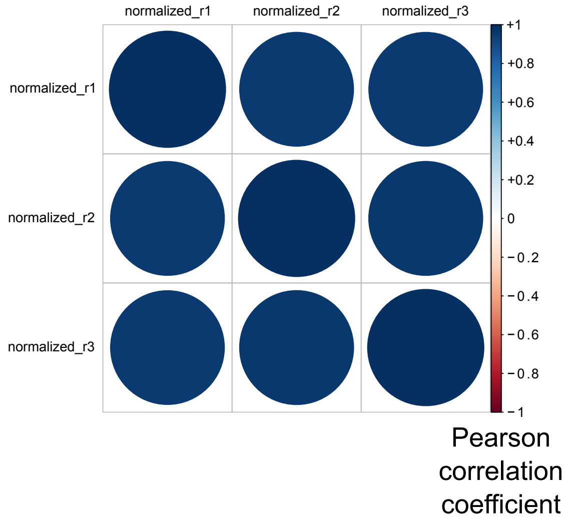

S9. Q-Q plot of morphology data: Sample and theoretical quantiles of negative controls (blue), positive controls (red) and samples from the mimic library (black), for eccentricity values.

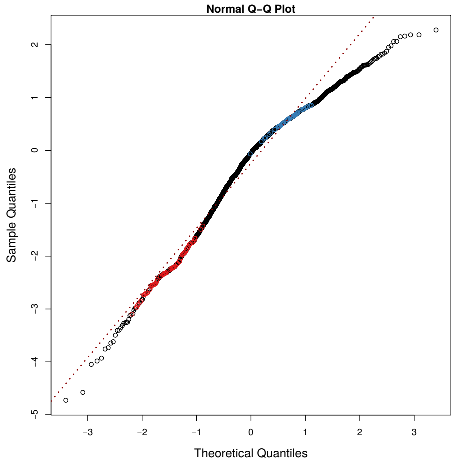

S10. Multi-feature analysis of morphology: Secondary features of morphology ("Area", "Compactness", "Eccentricity", "EulerNumber", "Extent", "FormFactor", "Solidity") were used on the traininig set to build a linear discriminant analysis model (A). It was applied to a test set, and the mosaic plot (B) shows low mis-classification rates using the model. The model was then applied to unknown samples to classify them into epithelial, intermediate, and mesenchymal morphology. All candidates, except one, classified as "epithelial" by the LDA model were also picked up as hits (Z-score < -1) by Eccentricity alone (C). Seed analysis of samples classified as "epithelial" shows an over-representation of the AAGUGC sequence (D), confirming the results from single-feature morphology classification (using Eccentricity alone - see Fig 6A)

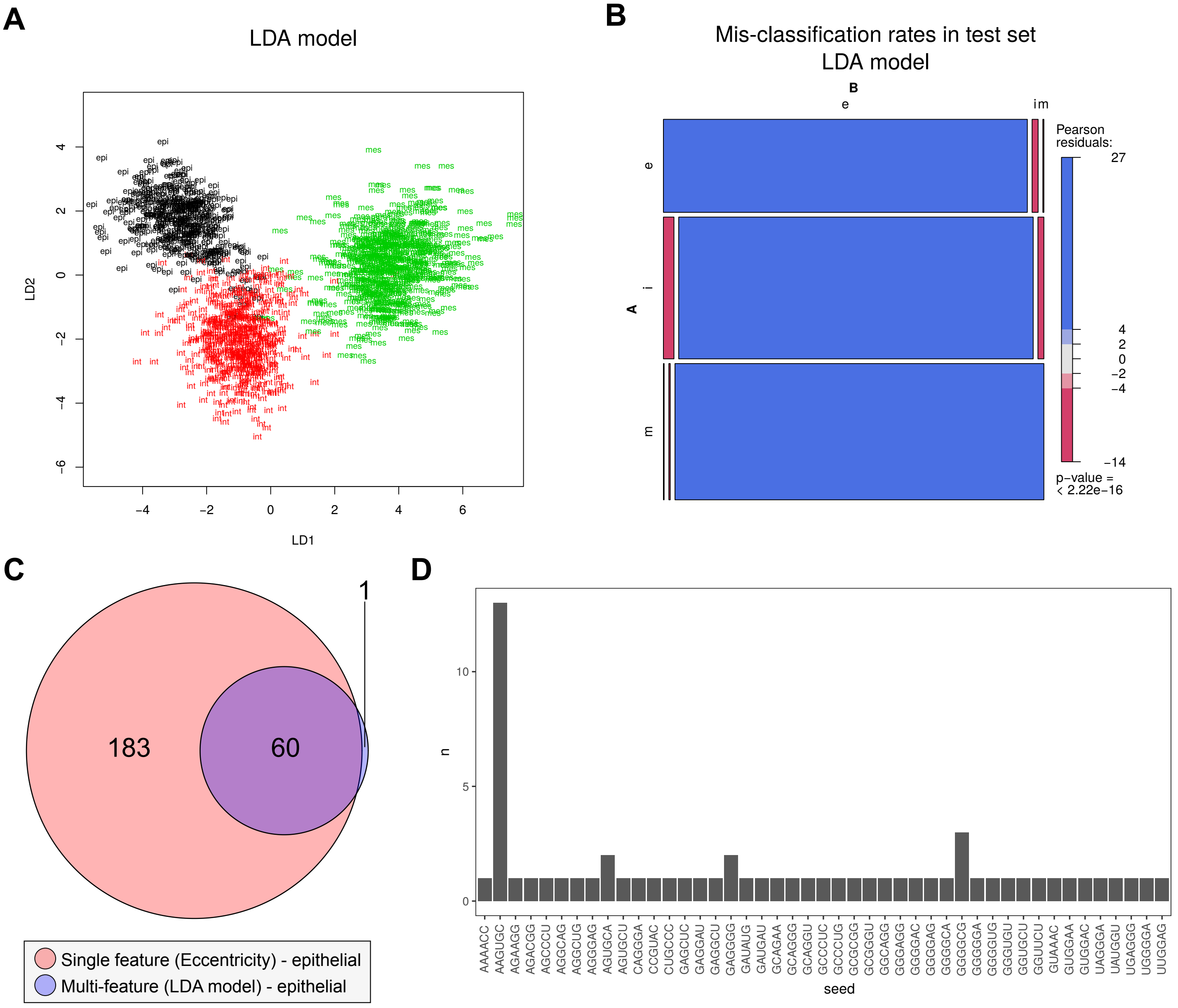

S11. RNA-seq analysis for microRNAs: MicroRNA counts for ARCaPE (e1-e3) and ARCaPM (m1-m3) prostate cancer cells was performed using Chimira and differential expression analysis was performed using DESeq2. Quality control in DESeq2 included count normalisation (left) and variance stabilisation (right).

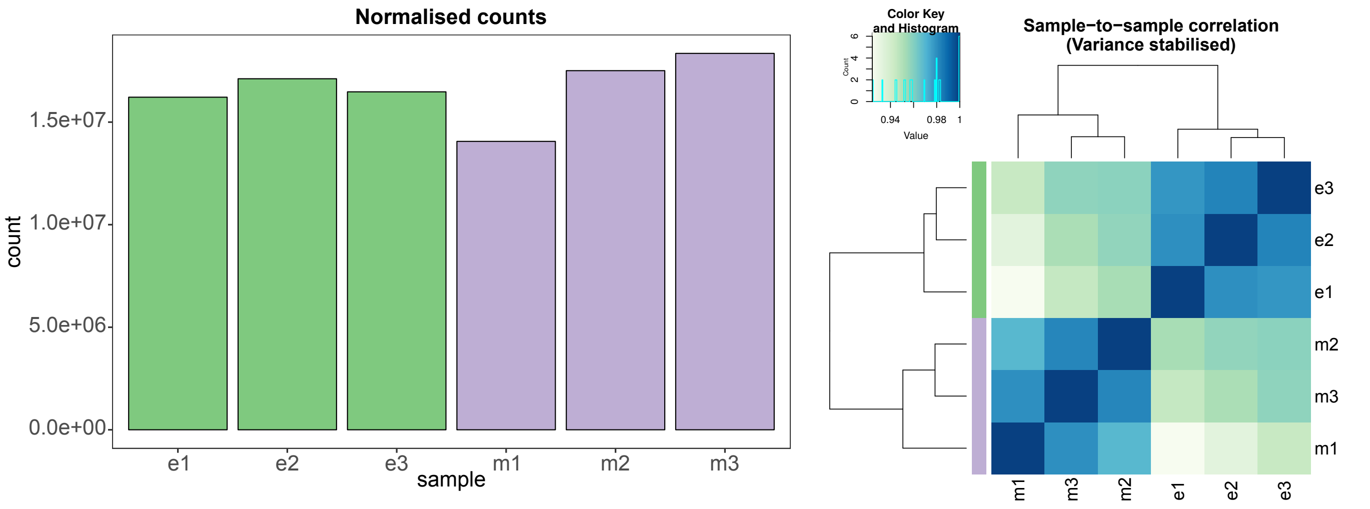

S12. AAGUGC motif: MicroRNAs containing the AAGUGC motif in positions 3-8 (from the 5' end) (A), differences in mean Z-scores between miRs containing the AAGUGC sequence at position 3-8 vs 2-7 (B), and all microRNAs containing the AAGUGC motif anywhere in their sequence (C).

A

logo

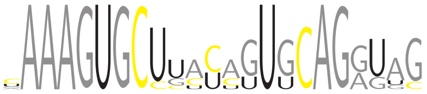

```
hsa-mir-106b-5p  UAAAGUGCUGACAGUGCAGAU..    21
hsa-mir-93-5p    CAAAGUGCUGUUCGUGCAGGUAG    23
hsa-mir-20b-5p   CAAAGUGCUCAUAGUGCAGGUAG    23
hsa-mir-20a-5p   UAAAGUGCUUAUAGUGCAGGUAG    23
hsa-mir-17-5p    CAAAGUGCUUACAGUGCAGGUAG    23
hsa-mir-106a-5p  AAAAGUGCUUACAGUGCAGGUAG    23
hsa-mir-519d-3p  CAAAGUGCCUCCCUUU.AGAGUG    22
hsa-mir-526b-3p  GAAAGUGCUUCCUUUU.AGAGGC    22
consensus         !!!!!!!******!**!!****
```

B

	Mean Z scores	
**Column1**	**Migration**	**Morphology**
**AAGUGC (3-8)**	0.23	-0.05
**AAGUGC (2-7)**	-1.03	-2.05

C

logo

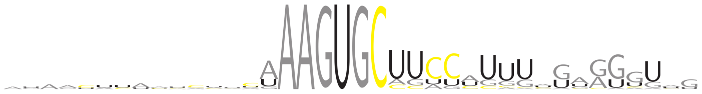

```
hsa-mir-302b-5p  ...ACUUUAACAUGGAAGUGCUUUC.............    22
hsa-mir-590-5p   .GAGCUUAUUCAUAAAAGUGCAG...............    22
hsa-mir-4263     ..........AUUCUAAGUGCCUUGGCC..........    18
hsa-mir-374c-5p  AUAAUACAACCUGCUAAGUGCU................    22
hsa-mir-18a-3p   .......ACUGCCCUAAGUGCUCCUUCUGG........    23
hsa-mir-512-3p   ...............AAGUGCUGUCAUAGCUGAGGUC.    22
hsa-mir-106b-5p  .............UAAAGUGCUGACAGUGC..AGAU..    21
hsa-mir-93-5p    .............CAAAGUGCUGUUCGUGC..AGGUAG    23
hsa-mir-20b-5p   .............CAAAGUGCUCAUAGUGC..AGGUAG    23
hsa-mir-20a-5p   .............UAAAGUGCUUAUAGUGC..AGGUAG    23
hsa-mir-17-5p    .............CAAAGUGCUUACAGUGC..AGGUAG    23
hsa-mir-106a-5p  .............AAAAGUGCUUACAGUGC..AGGUAG    23
hsa-mir-3616-5p  ............AUGAAGUGCACUCAUGAUAUGU....    22
hsa-mir-372-3p   ..............AAAGUGCUGCGACAUUUGAGCGU.    23
hsa-mir-371a-3p  ...............AAGUGCCGCCAUCUUUUGAGUGU    23
hsa-mir-302a-3p  ..............UAAGUGCUUCCAUGUUUUGGUGA.    23
hsa-mir-302b-3p  ..............UAAGUGCUUCCAUGUUUAGUAG.    23
hsa-mir-302d-3p  ..............UAAGUGCUUCCAUGUUUGAGUGU.    23
hsa-mir-302c-3p  ..............UAAGUGCUUCCAUGUUUCAGUGG.    23
hsa-mir-302e     ..............UAAGUGCUUCCAUGCUU.......    17
hsa-mir-373-3p   ..............GAAGUGCUUCGAUUUUGGGGUGU.    23
hsa-mir-519c-3p  ..............AAAGUGCAUCUUUUUAGAGGAU..    22
hsa-mir-519b-3p  ..............AAAGUGCAUCCUUUUAGAGGUU..    22
hsa-mir-520e     ..............AAAGUGCUUCCUUUUUGAGGG...    21
hsa-mir-526b-3p  .............GAAAGUGCUUCCUUUUAGAGGC...    22
hsa-mir-520f-3p  ...............AAGUGCUUCCUUUUAGAGGGUU.    22
hsa-mir-520c-3p  ..............AAAGUGCUUCCUUUUAGAGGGU..    22
hsa-mir-520b     ..............AAAGUGCUUCCUUUUAGAGGG...    21
hsa-mir-520d-3p  ..............AAAGUGCUUCUCUUUGGUGGGU..    22
hsa-mir-520a-3p  ..............AAAGUGCUUCCCUUUGGACUGU..    22
hsa-mir-519d-3p  .............CAAAGUGCCUCCCUUUAGAGUG...    22
hsa-mir-520g-3p  ............ACAAAGUGCUUCCCUUUAGAGUGU..    24
hsa-mir-520h     ............ACAAAGUGCUUCCCUUUAGAGU....    22
hsa-mir-519e-3p  ...............AAGUGCCUCCUUUUAGAGUGUU.    22
hsa-mir-519a-3p  ..............AAAGUGCAUCCUUUUAGAGUGU..    22
consensus                       !!!!!!**
```

X non conserved

X ≥50% conserved
